# Cancer incidence in the population exposed to dioxin after the "Seveso accident": twenty years of follow-up

**DOI:** 10.1186/1476-069X-8-39

**Published:** 2009-09-15

**Authors:** Angela Cecilia Pesatori, Dario Consonni, Maurizia Rubagotti, Paolo Grillo, Pier Alberto Bertazzi

**Affiliations:** 1 Unit of Epidemiology, Department of Preventive Medicine, Fondazione IRCCS Ospedale Maggiore Policlinico, Mangiagalli e Regina Elena, Via San Barnaba 8, 20122 Milano, Italy; 2 EPOCA Research Center, Department of Occupational and Environmental Health, Università degli Studi di Milano, Milan, Via San Barnaba 8, 20122 Milano, Italy

## Abstract

**Background:**

The Seveso, Italy accident in 1976 caused the contamination of a large population by 2,3,7,8-tetrachlorodibenzo-p-dioxin (TCDD). Possible long-term effects have been examined through mortality and cancer incidence studies. We have updated the cancer incidence study which now covers the period 1977-96.

**Methods:**

The study population includes subjects resident at the time of the accident in three contaminated zones with decreasing TCDD soil levels (zone A, very high; zone B, high; zone R, low) and in a surrounding non-contaminated reference territory. Gender-, age-, and period-adjusted rate ratios (RR) and 95% confidence intervals (95% CI) were calculated by using Poisson regression for subjects aged 0-74 years.

**Results:**

All cancer incidence did not differ from expectations in any of the contaminated zones. An excess of lymphatic and hematopoietic tissue neoplasms was observed in zones A (four cases; RR, 1.39; 95% CI, 0.52-3.71) and B (29 cases; RR, 1.56; 95% CI, 1.07-2.27) consistent with the findings of the concurrent mortality study. An increased risk of breast cancer was detected in zone A females after 15 years since the accident (five cases, RR, 2.57; 95% CI, 1.07-6.20). No cases of soft tissue sarcomas occurred in the most exposed zones (A and B, 1.17 expected). No cancer cases were observed among subjects diagnosed with chloracne early after the accident.

**Conclusion:**

The extension of the Seveso cancer incidence study confirmed an excess risk of lymphatic and hematopoietic tissue neoplasms in the most exposed zones. No clear pattern by time since the accident and zones was evident partly because of the low number of cases. The elevated risk of breast cancer in zone A females after 15 years since the accident deserves further and thorough investigation. The follow-up is continuing in order to cover the long time period (even decades) usually elapsing from exposure to carcinogenic chemicals and disease occurrence.

## Background

2,3,7,8-tetrachlorodibenzo-*p*-dioxin (TCDD), the most toxic congener in the family of polychlorinated dibenzo-dioxins, PCDD, is a nearly ubiquitous contaminant of the environment in which we live [1]. Potential health effects of TCDD have been investigated in high exposure circumstances such as, for example, manufacture and agricultural use, war, and industrial/environmental accidents. The International Agency for Research on Cancer and the US Environmental Protection Agency (EPA), classified TCDD as human carcinogen [2,3]; still the scientific debate persists on the actual cancer risk posed by TCDD to the general population [4-7]. The industrial accident that occurred in the Seveso, Italy area on July 10, 1976 exposed a large residential population to substantial amounts of TCDD. In the immediate aftermath, typical effects of exposure to polychlorinated hydrocarbons such as chloracne were observed mainly in children who were outdoors at the time the accident occurred [8]. A variety of other early and mid-term health effects were then investigated including reproductive, immunologic, metabolic changes with no clear indications of adverse outcomes [9]. Long term effects were investigated by means of mortality and cancer incidence studies [10]. The clearest and most consistent result in the mortality study after 25 years (1976-2001) was an excess of lymphatic and hematopoietic neoplasms in the most exposed groups living in zones A and B [11]. We report here the results of the five-year extension (1992-1996) of the cancer incidence study now covering the period 1977-1996. The cancer incidence study, although limited to a shorter follow-up period in comparison to the mortality study, has the clear advantages to use more accurate cancer diagnoses based on clinical data collection and to allow earlier detection of low lethality cancers.

## Methods

Methods used to identify the study population, exposure definition, follow-up and case ascertainment were previously described in detail [12] and are here briefly summarized.

### Exposure

**Figure 1:**
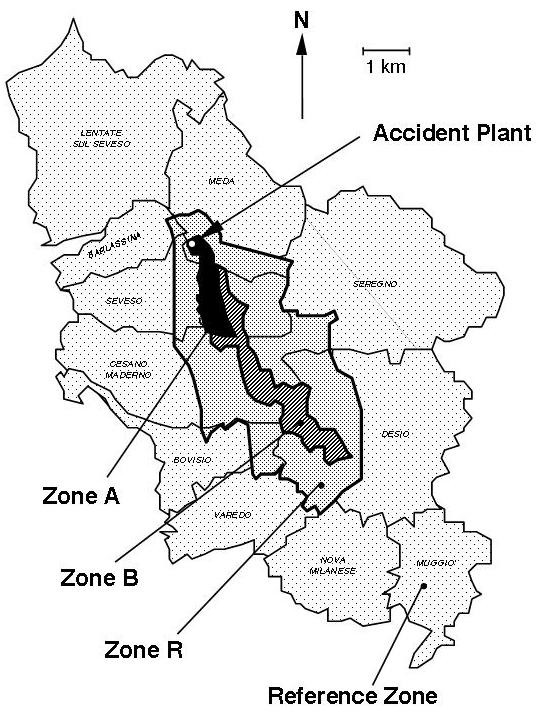
**Map of the Seveso, Italy area, including the territory of 11 towns**. The map indicates the three dioxin-contaminated zones with decreasing mean soil levels (A, B, and R) and the surrounding non-contaminated zone adopted as the reference.

The area where the toxic cloud released by a chemical factory deposited was subdivided into three zones based on measurements of TCDD soil levels [13]: Zone A (the most heavily contaminated), zone B (medium exposure) and zone R (low exposure); a surrounding non-contaminated territory including 11 municipalities was adopted as reference (figure 1).

Only ten years later, improvements in analytical techniques allowed to measure individual TCDD levels in the very small blood samples collected at the time of the accident from subjects chosen as the supposedly most exposed in zone A, B and R [14] and properly stored. Additional measurements were then performed in 110 subjects randomly selected from zone A and B and the non-contaminated reference zone in the early nineties [15]. As shown in Table 1, the zone-based classification of exposure was in fair agreement with blood TCDD measurements. The serum levels of six other PCDDs, 10 PCDFs, and four coplanar PCBs were also measured in these subjects [15]. We calculated total TEQ (Toxic Equivalency, the sum of congener-specific TEQs) using WHO-2005 TEFs (Toxic Equivalency Factors) [16]. In the contaminated zones none of the 20 congeners was above background values [15]. In particular: octa-chloro dioxin and furan (OCDD, OCDF) showed some variation across zone (p = 0.09 and p = 0.03, respectively; Kruskal-Wallis test), but their levels were lower than in the reference zone; for all the other congeners p-values were above 0.14. Total TEQ was clearly elevated in the polluted zones, whereas when we excluded TCDD from TEQ calculation no differences were found across zones (Table 1).

**Table 1:** TCDD soil measurements, serum TCDD and TEQ levels in residents in the Seveso area.

Zone	Mean soil TCDD **(μg/m^2^)**^a^	Serum TCDD (ppt)		**Serum TEQ**^b^ (ppt)	**Serum TEQ**^b^ without TCDD (ppt)
	**Min - Max**	**No.** **Subjects**	**Median**	**Median (Min - Max)**	**Median (Min - Max)**

**A**	15.5 - 580.4	296	447.0^c^	-	-
		7	73.3^d^	94.0 (60.6 - 141.7)	39.0 (16.7 - 51.8)

**B**	1.7 - 4.3	80	94.0^c^	-	-
		51	12.4^d^	43.2 (17.7 - 194.3)	31.8 (11.4 - 131.7)

**R**	0.9 - 1.4	48	48.0^c^	NA	NA

**Reference**	NA	52	5.5^d^	38.8 (10.2 - 92.1)	32.3 (8.2 - 82.4)

				p < 0.0001^e^	p = 0.92^e^

NA, not available

^a^Reference [13]

^b^Calculated using TEF WHO-2005 [16]; includes seven dioxins, 10 furans, four coplanar PCBs.

Levels below detection limit (DL) were set to DL/√2

^c^Blood samples collected in 1976 (reference [14])

^d^Blood samples collected in 1993-94 (reference [15])

^e^Kruskal-Wallis test

The median half-life of TCDD in serum in this population was estimated equal to 7.8 years with a longer half-life in women (nine years) than in men (seven years) [14]. Shorter half-lives in younger subjects (less than 18 years) [17] and in highly exposed adults [18] have been recently calculated. This would imply lower cumulative exposures in subjects with elevated TCDD concentrations.

Living in the area after the exposure mitigation and reclamation works did not seem to entail additional exposure: in a small sample of subjects entering zone B in the post-accident period serum TCDD levels were undetectable; in addition, sequential serum TCDD measurements in subjects residing in zone B at the time of the accident did not show increasing levels over time [14].

### Study population

The Seveso cohort includes all subjects living in one of the contaminated or reference zones at the date of the accident (July 10, 1976) and those who migrated into (or newborn in) the area in the 10-year period after the accident. Subjects were assigned to one of the contaminated or reference zones on the basis of their residence at the date of the accident or at entry into the area. About 80% of the cohort subjects were resident in the study area at the day the accident occurred. Table 2 reports their distribution by area and gender. The results reported here refer to the experience of the subgroup of subjects, aged 0-74 years, and living in the study area at the accident time.

**Table 2:** Number of subjects resident at the accident time (July 10, 1976) in the Seveso area.

Zone	Females	Males	Total
**A**	371	352	723

**B**	2,350	2,471	4,821

**R**	15,928	15,715	31,643

**Reference**	93,225	88,349	181,574

**Total**	111,874	106,887	218,761

### Follow-up and case ascertainment

The study population (exposed and non-exposed) has been followed up for mortality and cancer incidence as a unique cohort, with the same methods, blinded of the exposure status. Persons who moved outside the study area were nonetheless traced with a higher than 99% success rate [11].

Cancer cases ascertainment encompassed the 120 hospital-network of the Lombardy region where the study area is located. It is the most populated region of Italy (9,032,554 people out of 56,995,744 in the whole country at 2001 census). About 95% of the population was still residing within the region at the end of 1996. For case ascertainment, the complete information on all hospital admission/discharge forms (anonymous) in the Lombardy Region in the relevant period were linked with the records of cohort members using data on gender, date of birth and residence. This allowed the identification of the potential study subjects admitted in or discharged by a Lombardy hospital with a diagnosis mentioning cancer. Original medical records were then reexamined to identify true cases, to retrieve a diagnosis as accurate as possible and the actual date of occurrence. The number of individual medical files identified in the period 1992-1996 was 36,589 and 99.9% of them were successfully reviewed. In the absence of a region-wide cancer registration system, case ascertainment had to be performed individually, on medical papers, by ad hoc trained researchers. The study covered malignant tumours at any site, plus benign tumours of liver, bladder and central nervous system first diagnosed after the date of the accident. Cancer sites and morphology were coded using the International Classification of Diseases in use at the time of cancer occurrence and the International Classification of Diseases for Oncology (ICD-O) respectively.

Emigration was homogeneous across zones. The proportions of subjects who moved outside Lombardy were 5.8%, 6.7%, 4.7% for the exposed zones (A, B, R) and 5.6% for the reference zone. For subjects without hospitalization and people emigrated outside Lombardy cancer cases were identified solely from death certificates (Death Certificate Only, DCO).

### Statistical analysis

Rate ratios (RR) and 95% confidence intervals (95% CI) for zones A, B, R *vs *the reference zone were calculated using Poisson regression models adjusting by gender, age category, and period (five-year classes). Analysis by time since the date of the accident was also performed (0-4, 5-9, 10-14, 15+ years). The subgroup of subjects with a previous diagnosis of chloracne was separately examined. Only malignant tumours have been examined. All statistical analyses were performed using Stata, version 10 [19].

## Results

The total number of cases detected in the zones affected by dioxin contamination (A, B and R) was 2,122 and 660 (31.1%) occurred after 1991. The proportion of cases diagnosed through death certificate only (DCO) in the whole period was 7.2% and did not vary across zones. The overall histology confirmation rate over the study period is 82% with a slightly higher proportion in zone B (87%).

Incidence findings for specific cancer sites are reported in Table 3 by exposure zone. Overall, cancer incidence did not depart from expectations.

**Table 3:** Results of cancer incidence analyses in the Seveso population*, 1977-96.

Cancer sites (ICD-9 code)	Zone A (high exposure)			Zone B (medium exposure)			Zone R (low exposure)		
	**N**	**RR**	**95% CI**	**N**	**RR**	**95% CI**	**N**	**RR**	**95% CI**

All cancers (140-208)	44	1.03	0.76-1.38	270	1.00	0.89-1.13	1808	0.96	0.91-1.00

Digestive (150-159)	7	0.59	0.28-1.23	79	1.06	0.85-1.33	495	0.94	0.85-1.03

Esophagus (150)	0	-	-	1	0.26	0.04-1.91	35	1.33	0.92-1.92

Stomach (151)	3	0.86	0.28-2.69	19	0.87	0.55-1.37	131	0.84	0.70-1.01

Colon (153)	2	0.68	0.17-2.72	19	1.04	0.66-1.64	137	1.04	0.87-1.26

Rectum (154)	0	-	-	17	1.78	1.09-2.88	71	1.05	0.82-1.35

Liver (155)	0	-	-	14	1.29	0.76-2.20	56	0.74	0.56-0.97

Biliary tract (156)	0	-	-	6	2.28	1.00-5.17	16	0.82	0.49-1.39

Pancreas (157)	1	1.15	0.16-8.19	3	0.56	0.18-1.74	38	0.99	0.70-1.40

Other digestive (159)	1	6.96	0.96-50.6	0	-	-	3	0.46	0.14-1.48

Respiratory (160-165)	7	0.88	0.42-1.85	48	0.98	0.73-1.30	350	1.02	0.91-1.15

Lung (162)	7	1.12	0.53-2.36	37	0.96	0.69-1.33	280	1.04	0.92-1.19

Pleura (163)	0	-	-	4	3.38	1.22-9.37	5	0.60	0.24-1.50

Soft tissue sarcoma (171)	0	-	-	0	-	-	9	1.32	0.64-2.73

Soft tissue and visceral sarcomas	0	-	-	3	0.82	0.26-2.58	24	0.98	0.64-1.51

Melanoma (172)	1	1.62	0.23-11.61	2	0.50	0.12-2.03	19	0.71	0.44-1.14

Skin (173)	3	1.39	0.45-4.32	5	0.37	0.15-0.90	88	0.93	0.75-1.17

Breast (174)	8	1.43	0.71-2.87	30	0.85	0.59-1.22	249	1.00	0.88-1.15

Genito-urinary tract (179-189)	8	1.07	0.53-2.14	46	0.98	0.73-1.31	302	0.91	0.81-1.03

Uterus (179-182)	4	2.34	0.87-6.27	10	0.93	0.49-1.73	61	0.79	0.60-1.03

Cervix (180)	2	2.67	0.66-10.77	7	1.47	0.69-3.12	28	0.84	0.57-1.25

Endometrium (182)	1	1.24	0.17-8.82	3	0.60	0.19-1.87	27	0.73	0.49-1.10

Ovary (183)	1	1.11	0.16-7.90	1	0.18	0.02-1.25	45	1.12	0.82-1.54

Prostate (185)	0	-	-	7	0.94	0.45-1.99	39	0.75	0.54-1.05

Testis (186)	0	-	-	2	0.82	0.20-3.32	22	1.44	0.90-2.31

Bladder (188)	3	1.44	0.46-4.49	17	1.33	0.82-2.16	84	0.94	0.75-1.19

Kidney (189)	0	-	-	6	0.87	0.39-1.96	43	0.90	0.65-1.24

Brain (191)	2	2.43	0.60-9.79	4	0.76	0.28-2.045	37	1.04	0.73-1.48

Thyroid (193)	1	2.63	0.37-18.86	4	1.60	0.59-4.36	19	1.15	0.70-1.89

Lymphatic and hematopoietic tissue (200-208)	4	1.39	0.52-3.71	29	1.56	1.07-2.27	121	0.96	0.79-1.16

All lymphoma (200-202)	1	0.62	0.09-4.41	15	1.43	0.86-2.40	72	1.02	0.80-1.32

Hodgkin's disease (201)	0	-	-	3	1.20	0.38-3.78	23	1.46	0.91-2.29

Non-Hodgkin's lymphoma (200, 202)	1	0.80	0.11-5.69	12	1.51	0.85-2.69	49	0.90	0.66-1.22

Multiple myeloma (203)	1	2.88	0.40-20.70	6	2.77	1.2-6.32	18	1.15	0.70-1.91

Leukemia (204-208)	2	2.18	0.54-8.76	8	1.35	0.66-2.73	31	0.77	0.53-2.12

Lymphatic leukemia (204)	1	2.78	0.39-19.9	0	-	-	13	0.83	0.46-1.48

Myeloid leukemia (205)	1	2.23	0.31-15.99	7	2.41	1.12-5.18	15	0.76	0.44-1.30

Leukemia, unspecified (208)	0	-	-	1	2.16	0.29-16.10	2	0.61	0.14-2.60

*Subjects aged < 75 years, resident in the area at the accident time.

ICD-9: International Classification of Diseases, Ninth Revision; N: number of cases; RR: rate ratios calculated with Poisson regression, adjusted for gender, age category, and period; 95% CI: 95% confidence interval.

In zone A, sparse increased risks were found for multiple sites (skin, bladder, brain), however based on a very small number of cases. Seven lung cancer cases yielded a 10% increased risk; all cases were in males (RR, 1.25; 95% CI, 0.6-2.6). A 40% non-significant increase of breast cancer and a higher than two-fold non-significantly increased risk for uterus cancer were observed among females. One of the breast cancers was detected in a male (0.05 were expected). A moderate, non-significantly increased RR was also observed for neoplasms of the lymphohemopoietic tissues. In an attempt to distinguish pre- and post-menopausal cases of breast cancer, a separate analysis for cancer diagnosed before and after 50 years of age was done: the RRs were 1.50 (three cases, 95% CI, 0.48-4.67) and 1.39 (five cases, 95% CI, 0.58-3.36), respectively. All cases occurred in women aged 20-49 years at the time of the accident (RR, 1.98; 95% CI, 0.99-3.96).

In zone B, a 78% excess risk was found for rectal cancer. The excess was limited to males (13 cases; RR, 2.1; 95% CI, 1.2-3.7). A higher than two-fold increased risk was observed for cancers of the biliary tract. Of the six cases, four occurred among females yielding a RR of 3.1 (95% CI, 1.1-8.6). The excess risk was already present in the 15 years post accident analysis (1977-1991) and no additional cases were detected in this extended follow-up 10. Among respiratory cancers a three-fold significant increase was seen for pleural cancer, particularly among males (three cases; RR, 3.89; 95% CI, 1.19-12.7). Lymphohemopoietic neoplasms showed as a single category a 56% excess with borderline statistical significance. Multiple myeloma and myeloid leukaemia occurrence was clearly in excess.

Modest, non-significant increases were observed in zone R for esophageal cancer, testis cancer and Hodgkin's disease.

No cases of soft tissue sarcoma (ICD-9: 171) occurred in zone A and B (1.17 expected), whereas nine cases were observed in zone R yielding a 30% non-significant excess: seven cases occurred among males (RR, 2.1; 95% CI, 0.9-5.1) and two among females (RR, 0.6; 95% CI, 0.1-2.4). When also sarcomas of parenchymal organs were added, no increased risks were detected in any of the exposed zones (zone A: no cases; zone B: three cases and zone R: 24 cases).

Table 4 shows results of the analysis by time since the accident, for selected cancer causes.

**Table 4:** Results of cancer incidence analyses in the Seveso population*, 1977-96, by time since the accident.

Cancer sites	Zone		Years since the accident			
			**0-4**	**5-9**	**10-14**	**15 +**

All cancers	A	N	8	8	9	19
		RR	1.06	0.84	0.83	1.27
		95% CI	0.53-2.12	0.42-1.68	0.43-1.60	0.81-2.00

	B	N	55	51	72	92
		RR	1.13	0.84	1.05	1.02
		95% CI	0.87-1.48	0.63-1.10	0.83-1.32	0.83-1.26

	R	N	339	411	455	603
		RR	0.94	0.93	0.96	0.99
		95% CI	0.84-1.06	0.84-1.03	0.87-1.06	0.90-1.07

Lung cancer	A	N	1	1	1	4
		RR	0.84	0.73	0.57	2.04
		95% CI	0.12-5.96	0.10-5.19	0.08-4.08	0.76-5.47

	B	N	8	8	9	12
		RR	1.02	0.89	0.86	1.09
		95% CI	0.50-2.05	0.44-1.79	0.44-1.66	0.61-1.93

	R	N	37	70	83	90
		RR	0.66	1.09	1.15	1.20
		95% CI	0.47-0.92	0.84-1.41	0.91-1.46	0.95-1.50

Lymphatic and hematopoietic tissue cancer	A	N	-	-	1	3
		RR			1.39	2.96
		95% CI			0.20-9.96	0.95-9.22

	B	N	9	5	9	6
		RR	2.39	1.33	1.92	0.95
		95% CI	1.22-4.69	0.55-3.25	0.98-3.75	0.42-2.12

	R	N	20	27	32	42
		RR	0.75	1.03	1.01	1.00
		95% CI	0.47-1.20	0.68-1.55	0.70-1.48	0.72-1.38

Non-Hodgkin's lymphoma	A	N	-	-	-	1
		RR				1.97
		95% CI				0.27-14.07

	B	N	2	2	5	3
		RR	1.75	1.37	2.30	0.94
		95% CI	0.43-7.20	0.34-5.62	0.93-5.66	0.30-2.96

	R	N	5	15	8	21
		RR	0.61	1.44	0.54	0.99
		95% CI	0.24-1.53	0.82-2.53	0.26-1.12	0.62-1.58

Leukemia	A	N	-	-	1	1
		RR			5.11	3.26
		95% CI			0.71-37.07	0.45-23.44

	B	N	3	1	2	2
		RR	1.90	0.85	1.55	1.05
		95% CI	0.60-6.05	0.12-6.15	0.38-6.37	0.26-4.29

	R	N	6	8	14	3
		RR	0.55	0.98	1.60	0.24
		95% CI	0.24-1.26	0.46-2.07	0.88-2.90	0.07-0.75

Multiple myeloma	A	N	-	-	-	1
		RR				8.35
		95% CI				1.14-61.31

	B	N	2	2	2	-
		RR	3.56	4.76	4.47	
		95% CI	0.85-15.00	1.11-20.38	1.04-19.20	

	R	N	1	2	4	11
		RR	0.23	0.63	1.28	2.24
		95% CI	0.03-1.70	0.15-2.68	0.44-3.77	1.11-4.49

Breast cancer (females only)	A	N	-	1	2	5
		RR		0.81	1.42	2.57
		95% CI		0.11-5.74	0.35-5.68	1.07-6.20

	B	N	4	6	10	10
		RR	0.70	0.79	1.09	0.78
		95% CI	0.26-1.87	0.35-1.76	0.58-2.04	0.42-1.46

	R	N	48	59	55	87
		RR	1.10	1.07	0.87	1.01
		95% CI	0.81-1.49	0.81-1.41	0.66-1.15	0.81-1.27

*Subjects aged < 75 years, resident in the area at the accident time.

ICD-9: International Classification of Diseases, Ninth Revision; N: number of cases; RR: rate ratios calculated with Poisson regression, adjusted for gender and age category; 95% CI: 95% confidence interval.

In zone A, all cancers showed a slightly increased risk after 15 years. A similar pattern was observed for lung cancer, lymphohemopoietic neoplasms and breast cancer (significant after 15 years). In zone B, no definite time-related patterns were seen for all cancers and lung cancer. The most notable finding was the excess for lymphohemopoietic neoplasms observed in the 0-4 and 10-14 years categories (nine cases, RR, 2.39; 95% CI, 1.22-4.68 and nine cases, RR, 1.92; 95% CI, 0.98-3.75 respectively). Steadily increased risks for multiple myeloma were observed in each category within 15 years since the accident.

In zone R, a numerical increase of the RR values with time since initial exposure was observed: however, none of the values was significantly above unity with the exception of multiple myeloma after 15 years since the accident.

No other distinct patterns or trends were seen for other specific cancer causes (results not shown).

No cancer cases were observed among the group of people (n = 183) who were diagnosed as chloracne cases shortly after the accident. It's important to mention that subjects with chloracne were very young at the time of the accident (their mean age was 10 years). The age standardized number of expected cancer cases was 1.7.

## Discussion

The follow-up of the population affected by the Seveso accident in 1976 had the primary goal to identify possible late occurring consequences of exposure to TCDD on health. It also represented a unique opportunity to improve our present knowledge on the carcinogenic hazard posed by TCDD to human populations. In fact, both environmental [20] and biological data (serum TEQ without TCDD were similar across zones, as reported in Table 1) showed that TCDD was the only congener to which people in Seveso were exposed.

In animal models, TCDD is a multisite carcinogen that induces cancer in different organs, species and strains. Increased incidence of lymphomas, fibrosarcomas and neoplasms of liver, lung, thyroid, skin, tongue, hard palate and nasal turbinates have been found [21]. TCDD is generally characterized as a non-genotoxic carcinogen, a potent promoter and a weak initiator. Several potential mechanisms for carcinogenicity have been implicated including oxidative stress, indirect DNA damage, endocrine disruption, altered signal transduction and cell replication leading to tumour promotion [22]. The human epidemiologic evidence mainly relies on four industrial cohorts [23-26] with high exposures which showed a consistent increase in all cancers combined with a positive exposure-response trend. In interpreting these results, we need to consider recent studies which applied new models to estimate exposure in these cohorts and raised questions on the potential overestimate of the dose-response relationship [18,27]. Increased risks for some distinct cancer sites (lung, Non-Hodgkin's Lymphoma, soft tissue sarcoma) have also been reported but their specific association with TCDD exposure is less compelling [2].

Epidemiological studies, which are observational by nature, might be affected by several sources of bias. Some can be addressed in the design and conduct phases of the study, and some can only be indirectly addressed. Throughout the follow-up period, all tracing and case ascertainment procedures were implemented concurrently, with the same methods, and blinded of the exposure status of the subject for both the index and the reference population. Tracing for hospital admissions was conducted within the Lombardy region where 95% of the study population was still residing at the end of the follow-up. Emigration rates outside Lombardy were low and similar across zones, thus minimizing the possible bias due to exposure related selective migration. A definite limitation of our study is the exposure categorization which was based on environmental contamination data (TCDD soil measurements) and the official residence of the subjects at the time of the accident. In the absence of individual exposure data, misclassification of exposure might have occurred since the level of exposure inside each zone was not homogeneous and could vary considerably; moreover, official residence does not necessarily coincide with actual presence in the area at the time of the accident. Any such misclassification should be non-differential with risk estimates biased towards the null. Importantly, the extent of such possible misclassification is attenuated by further pieces of information available. Later TCDD blood measurements, although in limited samples, lent credibility to the existing zone categorization (A very high, B high, R low and scanty) and also showed that in the reference zone people exposure levels were similar to the published background values [15]. Also, data collected through questionnaire in cross-sectional studies in the area showed that official residence is highly concordant with presence in the area at the time of the accident [15]. The index and reference populations are included within the same health district and share major macro and micro-environmental factors - including health services, referral physicians, life style, industrial and occupational features, diet and leisure. This close comparability provides fair assurance of an indirect control of other major, relevant and possibly confounding risk factors.

The study confirmed the excess of lymphatic and hematopoietic neoplasms although without a clear pattern across zones of decreasing average exposure. The finding is consistent with the results of the concurrent mortality study [11] where, in addition, an exposure related risk pattern was visible. The small number of events and the lack of individual exposure metrics may have affected the results. The increase was visible, for the first time, also in the small yet highly polluted zone A particularly after 15 years since the accident, whereas in zone B the risk was high in the early post-accident period. The increase is consistent with the findings of occupational cohort studies [23-25,28] and with experimental data [21]. In addition, a cohort of Finnish fishermen with dioxin concentrations comparable to those found in the Seveso population, showed a 28% non-statically significant increased mortality from lymphatic and hematopoietic neoplasms [29]. Comparisons by specific lymphohemopoietic neoplasms across studies are made difficult by the small number of events.

The slightly increased risk for breast cancer in zone A females became significant after 15 years since the accident, based on five cases. No such increase was detected in zone B. This finding is consistent with the Seveso Women's Health Study that showed a dose response relationship between breast cancer and serum TCDD levels in the highly exposed women resident in zone A and B at the time of the accident after adjusting for other major risk factors such as parity, lactation, age at first pregnancy, smoking, etc [30]. Industrial cohorts were mainly comprised of men; the most updated mortality of the IARC international cohorts showed a twofold increased risk for breast cancer among female workers exposed to TCDD or higher chlorinated dioxins [28]. The increase was restricted to the only cohort with a consistent portion of female workers [31]. A mortality study conducted in Russia reported a two-fold increased risk of breast cancer among women living in Chapaevsk, an area contaminated by dioxin by a chemical plant producing exachlorocycloexane and its derivatives [32]. In addition, although limited by the ecological nature of the study, a spatial correlation between increased breast cancer incidence and soil dioxin contamination in a few areas in Michigan, USA, has been described [33]. TCDD is known to have some anti-estrogenic effects, however accumulating evidence suggests that TCDD also possesses estrogen-like activities. In particular, it has been suggested that the anti-estrogenic effects in the presence of estrogen and the estrogenic effects in its absence may alter the effects of TCDD depending on life stage at exposure [34]. All women with breast cancer in zone A were exposed to the accident between 20-49 years and the risk did not differ for pre- and post-menopausal cancers.

In interpreting the results for gynecological tumors, chance cannot be excluded as a credible explanation of the noted increase. Few studies on TCDD exposed females exist, and the extension of the follow-up will probably provide some further clues for interpretation.

Among people living in zone A, a twofold increased, although statistically non-significant, risk for lung cancer (in males) was estimated, after a 15-year latency period. The concurrent and most extended mortality follow-up had already shown in this zone a 60-70% increased risk after 15 years of follow-up [11]. Slightly increased mortality from lung cancer has been found in the four most exposed industrial cohorts, particularly in highly exposed subjects, but when dose-response relationships were examined, some uncertainty remained about the nature of the association with TCDD exposure [23-26,35,36]. Confounding by smoking has been evaluated in most of these studies and could not entirely explain the observed excess. We did only indirectly control for smoking habits in this study, based on information collected from limited samples and on the documented social and cultural homogeneity of the groups compared in this study [37].

Soft tissue sarcomas have been repeatedly associated to dioxin exposure [2]. In our population no cases were observed in the most exposed zones (1.17 were expected). A modest non-significant increased risk was detected among males in the least exposed zone R. Overall our data provide us with poor evidence of the association between dioxin exposure and soft tissue sarcoma in agreement with a recent case-control study which failed to show an increased risk at comparable exposure levels [38].

The increased risk for biliary tract cancer among females in zone B was already present in the 15 year analysis. No new cases have been detected after 1991.

Two further distinctly increased risks in zone B should be considered. The rectal cancer increase we observed among males has not been clearly associated to TCDD exposure in other epidemiologic studies and is not supported by experimental data. The increase of pleural cancer occurrence is probably due to asbestos exposure well documented in two chemical plants located in the study area.

The absence of cancer cases among chloracne subjects can be explained in terms of small population size and youth of the subjects at the time of the accident.

## Conclusion

The Seveso population constitutes a unique opportunity to evaluate the carcinogenic risk posed by TCDD (the main congener to which the population was exposed). The five-year (1992-1996) extension of the cancer incidence study confirmed an excess risk of lymphatic and hematopoietic neoplasms in the most exposed population groups although no consistent pattern by time since the accident was evident. An elevated risk of breast cancer was noted in zone A after 15 years since the accident and it deserves further and thorough investigation. The follow-up is continuing in order to cover the long time period (even decades) usually elapsing between exposure to carcinogenic chemicals and disease occurrence. Overall, our findings support the evaluation that TCDD represents a carcinogenic hazard to exposed people, at least at the levels experienced by this population after an industrial accident.

## Abbreviations

TCDD: 2,3,7,8-tetrachlorodibenzo-*p*-dioxin; RR: rate ratios; 95% CI: 95% confidence interval; ICD-9: International Classification of Diseases, Ninth Revision; TEQ: Toxic Equivalency; TEF: Toxic Equivalency Factor; PCDDs: polychlorinated dibenzo-dioxins; PCDFs polychlorinated dibenzo-furans; PCBs: polychlorinated biphenyls; OCDD: octa-chloro dioxin; OCDF: octa-chloro furan.

## Competing interests

The authors declare that they have no competing interests.

## Authors' contributions

ACP wrote the manuscript, designed the study and directed its implementation, supervised field activities, and performed quality controls. DC performed final data management and statistical analysis. MR helped in reviewing clinical information, cancer diagnoses and coding activities. PG was responsible of record linkage for cancer cases ascertainment for a large part of the cohort. PAB coordinated the Seveso long-term study and contributed to the interpretation of results and writing of the manuscript. All authors read and approved the final manuscript.
